# Diabetes diagnosis and management among insured adults across metropolitan areas in the U.S.

**DOI:** 10.1016/j.pmedr.2018.03.014

**Published:** 2018-03-28

**Authors:** Wenya Yang, Timothy M. Dall, Eleonora Tan, Erin Byrne, William Iacobucci, Ritashree Chakrabarti, F. Ellen Loh

**Affiliations:** a The Lewin Group, 3130 Fairview Park Drive, Suite 500, Falls Church, VA 22042, USA; b IHS Markit, 1300 Connecticut Ave., NW, Suite 401, Washington, DC 20036, USA; c Novo Nordisk Inc., 800 Scudders Mill Road, Plainsboro, NJ 08536, USA; d The Peter Lamy Center on Drug Therapy and Aging, Department of Pharmaceutical Health Services Research, University of Maryland Baltimore, 220 Arch St, Room 12-212, Baltimore, MD 21201, USA

**Keywords:** Diabetes, Management

## Abstract

This study provides diabetes-related metrics for the 50 largest metropolitan areas in the U.S. in 2012—including prevalence of diagnosed and undiagnosed diabetes, insurance status of the population with diabetes, diabetes medication use, and prevalence of poorly controlled diabetes.

Diabetes prevalence estimates were calculated using cross-sectional data combining the Behavioral Risk Factor Surveillance System, American Community Survey, National Nursing Home Survey, Census population files, and National Health and Nutrition Examination Survey. Analysis of medical claims files (2012 de-identified Normative Health Information database, 2011 Medicare Standard Analytical Files, and 2008 Medicaid Analytic eXtract) produced information on treatment and poorly controlled diabetes by geographic location, insurance type, sex, and age group.

Among insured adults with diagnosed type 2 diabetes in 2012, the proportion receiving diabetes medications ranged from 83% in Oklahoma City, Oklahoma, to 65% in West Palm Beach, Florida. The proportion of treated patients with medical claims indicating poorly controlled diabetes was lowest in Minneapolis, Minnesota (36%) and highest in Texas metropolitan areas of Austin (51%), San Antonio (51%), and Houston (50%).

Estimates of diabetes detection and management across metropolitan areas often differ from state and national estimates. Local metrics of diabetes management can be helpful for tracking improvements in communities over time.

## Highlights

• Key diabetes metrics vary by MSA and differ substantially from the national averages.
• New York City had 1.2 million adults with diabetes; Southern New Jersey had 50,200.
• Undiagnosed diabetes rate ranged from 37% in Los Angeles to 24% in Nashville.
• Among the insured type 2 diabetes patients, medication use ranged from 83% to 65%.
• Patients with medical claims indicating uncontrolled diabetes ranged from 51% and 36%.

## 1 Introduction

Diabetes is a major epidemic in the United States, yet many people with diabetes are undiagnosed, uninsured, or have suboptimal health or adherence outcomes (Centers for Disease Control and Prevention, 2014a, Centers for Disease Control and Prevention, 2014b; Dall et al., 2016). Detection, insurance coverage and quality of care for people with diabetes were anticipated to improve with implementation of the Affordable Care Act (ACA), new U.S. Preventive Services Task Force guidelines around screening and treatment, and evolving standards of care (US Preventive Services Task Force, 2015; American Diabetes Association, 2017). The diabetes-related implications of changes in policy and treatment guidelines may take years to become apparent, but evaluating their impact over time requires baseline diabetes metrics for comparison.

Select metrics of diabetes detection and management have been calculated for 2012 at the state and national levels (Dall et al., 2016; Centers for Disease Control and Prevention, 2014a, Centers for Disease Control and Prevention, 2014b). In 2012, 8.1 million adults were unaware they had diabetes (CDC, 2014a) and another 4.9 million with diagnosed diabetes lacked medical insurance (Dall et al., 2016). About 92% of insured adults with diagnosed diabetes had type 2 diabetes, and many of these adults had suboptimal outcomes related to medication adherence, glycemic control, and presence of complications. For example, among insured type 2 patients receiving antidiabetic medications, the proportion with medical claims indicating poor diabetes control ranged from 53% in Texas to 29% in Minnesota and Iowa (Dall et al., 2016).

Limited information is available to assess type 2 diabetes detection and management across metropolitan areas, and the existing information is based primarily on self-reported information collected through telephone surveys (CDC, 2014b). This study extends previously published national and state analyses (Dall et al., 2016) to construct diabetes detection and management metrics for the 50 largest metropolitan areas where over half the nation's population with diabetes resided in 2012. The claims analysis focused on the diagnosed type 2 population with medical insurance. Such information provides local baseline metrics to track progress over time in diagnosing and treating people with type 2 diabetes.

## 2 Methods

Our approach to calculate type 2 prevalence, detection and treatment outcomes by metropolitan area is similar to the approach we used to model national and state metrics (American Diabetes Association, 2013; Dall et al., 2014a, Dall et al., 2014b, Dall et al., 2016). We first estimated the size of the adult population in each metropolitan area by age group (20–34, 35–44, 45–54, 55–59, 60–64, 65–70, and over 70 years), sex, and insurance type (private, Medicare, Medicaid, uninsured). We used survey data to estimate the prevalence of diagnosed and undiagnosed diabetes for each segment of the population (by demographic and insurance type), and used medical and pharmaceutical claims to estimate the proportion of diabetes patients with type 2 and the prevalence of treatment outcomes within each segment. We aggregated results across demographic groups to provide metropolitan-level statistics.

The metropolitan areas modeled are officially designated Metropolitan Statistical Areas (MSAs), with three exceptions: (1) New York-Newark-Jersey City, NY-NJ-PA MSA was split into the New York population and the Northern New Jersey metropolitan designation, (2) Orange County, California was carved out of the Los Angeles-Long Beach-Anaheim, California MSA and reported separately, and (3) West Palm Beach was reported separately from Miami-Fort Lauderdale, Florida (whereas the official designation of this metropolitan statistical area is Miami-Fort Lauderdale-West Palm Beach).

This study used secondary data sources and received an exemption from the New England Institutional Review Board.

### 2.1 Data sources and selection and exclusion criteria

To construct the population file for each MSA we started with data on population size by 5-year age group, sex and race/ethnicity (non-Hispanic white, non-Hispanic black, non-Hispanic other, Hispanic) from the 2012 U.S. Census Bureau Annual County Estimates Population file linked to each MSA (U.S. Census Bureau, 2015a, U.S. Census Bureau, 2015b). To construct a person-level file for each MSA, we used random selection with replacement from the 2012 American Community Survey (ACS, n = 2,375,715) to draw a sample of people living in a metropolitan area where the number of records drawn reflected the size of each demographic strata defined by 5-year age group, sex, and race/ethnicity among the ACS participants in each state. From the ACS data, we obtained information on household income, medical insurance status, and whether the person resides in the community or in a nursing home. Each person in the constructed population who resides in the community was then matched with a similar person from the 2011 and 2012 Behavioral Risk Factor Surveillance System (BRFSS, n = 982,154) using random sampling with replacement to match by age group, sex, family income level (8 levels), insurance status, metropolitan residency status, and state. For individuals in the ACS file who reside in a nursing home we used random sampling with replacement to match each ACS person with a nursing home resident in the 2004 National Nursing Home Survey (NNHS, n = 13,507) of similar age, sex, and race/ethnicity.

The resulting population file for each MSA contained a representative sample of the population complete with diagnosed diabetes prevalence; demographics; previous diagnosis or history of asthma, arthritis, heart attack, stroke, cancer, hypertension, high cholesterol, and cardiovascular disease; current smoker; body weight defined by body mass index (National Institutes of Health, 2000)—normal (BMI < 25), overweight (25 ≤ BMI < 30), or obese (30 ≤ BMI); and insurance type (Medicare, Medicaid, commercially insured, uninsured). Diagnosed diabetes prevalence, presence or history of the other chronic diseases modeled, and body weight information for the community-based population was self-reported in BRFSS. For the nursing home population, disease status and body weight information came from clinical diagnosis in NNHS. This information allowed us to estimate the prevalence of diagnosed diabetes by insurance type within each MSA.

Estimated prevalence of undiagnosed diabetes for each MSA was constructed by applying a regression-based predictive model (described later) to each person in the representative population sample. This predictive model was estimated using the 2005–2012 files of the National Health and Nutrition Examination Survey (NHANES) for adults without a previous diagnosis of diabetes and who did not use insulin. NHANES is a nationally representative sample of the non-institutionalized population. Approximately one third of NHANES adults were randomly chosen to undergo laboratory tests—including hemoglobin A1c (A1c) and fasting plasma glucose testing (FPG) that we used to determine diabetes, prediabetes, or normal glucose status. The sample analyzed excluded pregnant women (n = 463) and consisted of adults with previously undiagnosed diabetes (n = 1209), prediabetes (n = 7190), or normal glucose levels (n = 10,719).

Diabetes treatment outcomes were calculated using medical and pharmacy claims data for each population strata (e.g. insurance type, age group, and sex), and were multiplied with the population size in the corresponding strata from the constructed population file for each MSA. Medical claims for commercially insured adults in each MSA came from the 2011–2012 OptumInsight de-identified Normative Health Information database (dNHI, n = 29,948,496), for the Medicare population from the 2011 Medicare 5% sample (n = 2,805,812), and for the Medicaid population using the Centers for Medicare and Medicaid Services (CMS) 2008 Mini-Max file (n = 3,095,634). The dNHI database used medical and medication claims and membership data from January 2011 through December 2012, and the file contains longitudinally-linked and statistically de-identified individual-level data from UnitedHealth Group and non-UnitedHealth Group sources. The Medicare 5% sample contains medical and prescription claims. Mini-Max is a 5% sample of the Medicaid Analytic eXtract data—a set of person-level data files on Medicaid eligibility, service utilization, and payments for >60 million Medicaid enrollees extracted from the Medicaid Statistical Information System.

Patients analyzed in each of these databases were continuously enrolled in a fee-for-service coverage type plan with no more than one gap in enrollment of up to 45 days during the measurement year for the commercially insured population, and were enrolled for all 12 months for the Medicare and Medicaid populations.

### 2.2 Definitions of key outcomes

Key outcomes modeled include: undiagnosed diabetes, type 2 diabetes, receiving medication for diabetes, and poorly controlled diabetes.

#### 2.2.1 Undiagnosed diabetes patients

Patients in NHANES with blood glucose levels indicating diabetes but who had not previously been diagnosed by a health provider and were not taking insulin or oral anti-diabetic medication. Similar to the approach used by CDC (2017), we identified NHANES participants with undiagnosed diabetes as those with FPG ≥ 126 or A1c ≥ 6.5. A limitation of NHANES is no follow-up confirmatory test is available, which introduces false positives and negatives. The predictive model applied to the metropolitan population files to estimate undiagnosed diabetes prevalence has been described elsewhere (Dall et al., 2014a, Dall et al., 2014b, Dall et al., 2016). This model used logistic regression with diagnosis status as the dependent variable. Explanatory variables consisted of demographics; presence of diseases included in the constructed population files (e.g., hypertension, cardiovascular disease); overweight, obesity and current smoking status; and household income and insurance status.

#### 2.2.2 Type 2 diabetes

Within the three medical claims databases, we identified patients with diabetes if the patient had at least one emergency department visit or hospitalization or two ambulatory visits (30 days apart) with diabetes diagnosis (International Classification of Diseases, Ninth Revision, Clinical Modification [ICD-9-CM] diagnosis code of 250.xx) submitted during the year, or whether the patient used insulin or other diabetes-related medications. Sample inclusion and exclusion criteria, as well as the algorithm for distinguishing whether a patient had type 1 or type 2 diabetes based on medications taken and diagnosis codes, are described in detail elsewhere (Dall et al., 2016). The constructed analytic sample only included patients with diagnosed type 2 diabetes and excluded people younger than 18 and adults who had evidence of gestational diabetes or a pregnancy.

#### 2.2.3 Medically treated diabetes

We analyzed pharmacy claims to identify patients receiving treatment for their diabetes. Diabetes treatment was defined as having any pharmacy claims for insulin, non-insulin injectables, or oral antidiabetic agents. National Drug Code therapeutic classes were used to identify the medication (Dall et al., 2016).

#### 2.2.4 Poor control status

Whether a person's diabetes is under control is generally determined by blood glucose levels (ADA, 2017; Christophi et al., 2012), but lab results with A1c values were available only for a subset of the commercially insured patients in the dNHI database and were unavailable for the Medicare and Medicaid populations. To consistently identify patients with poorly controlled diabetes we used ICD-9 diagnosis codes of 250.x2 and 250.x3 in any claim. For discussion, we refer to poorly controlled status for anyone with an ICD-9 code indicating uncontrolled diabetes at some point during the year, and “controlled” status was determined by the lack of a code indicating “uncontrolled” status.

## 3 Results

### 3.1 Diabetes prevalence

Diabetes metrics for each MSA are presented in Table 1, Table 2 (levels) and Table 3 (percentages), with confidence intervals in Appendix Tables 1a–3a, respectively. Half (53%) of U.S. adults resided in these MSAs, including 51% of diabetes patients and 55% of adults with undiagnosed diabetes cases (Table 1). New York City had the largest number of adults with diabetes (1.2 million), while Los Angeles had the largest number with undiagnosed diabetes (366,200) and the largest number with diagnosed diabetes who lacked medical insurance (131,900) (Table 1).

**Table 1:** Diabetes population, diagnosis and insurance status (ranked by diabetes cases): adults age 20+, 2012.

Metropolitan area	Total diabetes	# undiagnosed diabetes	Diagnosed	
			# uninsured	# insured
New York-Newark-Jersey City, NY-NJ-PA (excluding Northern NJ)	**1,238,100**	361,300	89,800	**787,000**
Los Angeles-Long Beach-Anaheim, CA (excluding Orange County)	997,900	**366,200**	**131,900**	499,800
Chicago-Naperville-Elgin, IL-IN-WI	869,300	252,700	115,100	501,500
Northern NJ	644,400	189,200	59,400	395,800
Dallas-Fort Worth-Arlington, TX	574,400	161,400	94,900	318,100
Houston-The Woodlands-Sugar Land, TX	560,600	155,500	100,800	304,300
Philadelphia-Camden-Wilmington, PA-NJ-DE-MD	560,000	155,500	48,700	355,800
Miami-Fort Lauderdale, FL (excluding West Palm Beach, FL)	544,500	181,500	83,900	279,100
Washington-Arlington-Alexandria, DC-VA-MD-WV	530,400	153,100	60,400	316,900
Atlanta-Sandy Springs-Roswell, GA	491,800	130,400	75,300	286,100
Detroit-Warren-Dearborn, MI	439,900	114,900	46,800	278,200
San Francisco-Oakland-Hayward, CA	411,500	129,900	43,600	238,000
Boston-Cambridge-Newton, MA-NH	397,500	113,800	25,300	258,400
Phoenix-Mesa-Scottsdale, AZ	379,400	108,800	39,900	230,700
Riverside-San Bernardino-Ontario, CA	359,900	111,500	50,600	197,800
Tampa-St. Petersburg-Clearwater, FL	301,500	82,200	39,600	179,700
Seattle-Tacoma-Bellevue, WA	298,100	88,600	31,200	178,300
St. Louis, MO-IL	272,700	72,000	34,300	166,400
Orange County, CA	268,600	87,200	26,300	155,100
San Diego-Carlsbad, CA	261,500	83,100	31,500	146,900
Baltimore-Columbia-Towson, MD	261,000	73,500	21,400	166,100
Minneapolis-St. Paul-Bloomington, MN-WI	242,300	77,000	18,000	147,300
Pittsburgh, PA	229,700	63,300	17,800	148,600
Cleveland-Elyria, OH	228,900	54,900	25,200	148,800
San Antonio-New Braunfels, TX	225,600	60,500	41,400	123,700
Orlando-Kissimmee-Sanford, FL	212,600	59,400	32,200	121,000
Charlotte-Concord-Gastonia, NC-SC	211,800	55,500	33,300	123,000
Cincinnati, OH-KY-IN	209,200	50,600	25,700	132,900
Kansas City, MO-KS	189,200	49,300	24,500	115,400
Denver-Aurora-Lakewood, CO	189,000	60,700	20,800	107,500
Portland-Vancouver-Hillsboro, OR-WA	188,500	55,800	20,400	112,300
Sacramento-Roseville-Arden-Arcade, CA	180,300	56,900	19,000	104,400
Columbus, OH	179,600	44,500	21,000	114,100
Indianapolis-Carmel-Anderson, IN	175,800	45,900	24,200	105,700
Nashville-Davidson-Murfreesboro-Franklin, TN	170,400	41,000	26,500	102,900
Virginia Beach-Norfolk-Newport News, VA-NC	167,400	43,000	22,600	101,800
Las Vegas-Henderson-Paradise, NV	166,700	54,700	19,300	92,700
West Palm Beach, FL	158,700	46,000	14,600	98,100
Austin-Round Rock, TX	148,800	43,100	25,000	80,700
Memphis, TN-MS-AR	142,600	35,000	23,900	83,700
Providence-Warwick, RI-MA	139,100	39,800	11,900	87,400
Milwaukee-Waukesha-West Allis, WI	135,600	38,800	12,400	84,400
Jacksonville, FL	132,200	35,400	19,200	77,600
Oklahoma City, OK	129,100	32,300	21,500	75,300
Richmond, VA	125,500	32,000	16,500	77,000
Hartford-West Hartford-East Hartford, CT	103,100	31,500	7900	63,700
Raleigh, NC	102,800	27,400	16,800	58,600
New Haven-Milford, CT	73,400	22,600	5600	45,200
Salt Lake City, UT	73,000	22,500	7300	43,200
Southern NJ	**50,200**	**14,700**	**4300**	**31,200**
Total (50 metro areas)	**15,344,100**	**4,466,400**	**1,829,500**	**9,048,200**
Relative to national totals	**51%**	**55%**	**49%**	**49%**

Note: highest and lowest MSA estimates are bolded.

**Table 2:** Diabetes T2 DM population, treatment status, and diabetes control (ranked by diabetes cases): adults age 20+, 2012.

Metropolitan area	Type 2			
	# untreated with Rx	Treated with Rx		
		# treated	# poorly controlled	# controlled
New York-Newark-Jersey City, NY-NJ-PA (excluding Northern NJ)	**215,500**	**512,700**	**223,800**	**288,900**
Los Angeles-Long Beach-Anaheim, CA (excluding Orange County)	114,300	354,900	154,300	200,600
Chicago-Naperville-Elgin, IL-IN-WI	103,800	361,900	158,400	203,500
Northern NJ	105,900	256,100	114,500	141,600
Dallas-Fort Worth-Arlington, TX	59,300	236,400	110,200	126,200
Houston-The Woodlands-Sugar Land, TX	61,500	222,800	111,400	111,400
Philadelphia-Camden-Wilmington, PA-NJ-DE-MD	84,200	239,800	97,900	141,900
Miami-Fort Lauderdale, FL (excluding West Palm Beach, FL)	69,700	185,100	91,200	93,900
Washington-Arlington-Alexandria, DC-VA-MD-WV	66,200	223,600	105,700	117,900
Atlanta-Sandy Springs-Roswell, GA	58,600	206,700	95,000	111,700
Detroit-Warren-Dearborn, MI	79,000	181,600	82,900	98,700
San Francisco-Oakland-Hayward, CA	51,500	168,200	72,600	95,600
Boston-Cambridge-Newton, MA-NH	55,700	181,800	73,800	108,000
Phoenix-Mesa-Scottsdale, AZ	70,600	143,300	66,100	77,200
Riverside-San Bernardino-Ontario, CA	38,700	143,800	65,600	78,200
Tampa-St. Petersburg-Clearwater, FL	43,400	124,700	58,000	66,700
Seattle-Tacoma-Bellevue, WA	34,800	129,700	53,800	75,900
St. Louis, MO-IL	33,600	120,900	55,300	65,600
Orange County, CA	33,800	109,500	47,700	61,800
San Diego-Carlsbad, CA	30,000	105,400	40,800	64,600
Baltimore-Columbia-Towson, MD	37,900	116,600	55,600	61,000
Minneapolis-St. Paul-Bloomington, MN-WI	29,500	103,500	37,100	66,400
Pittsburgh, PA	33,500	104,500	43,200	61,300
Cleveland-Elyria, OH	32,000	104,900	45,500	59,400
San Antonio-New Braunfels, TX	25,700	92,600	47,000	45,600
Orlando-Kissimmee-Sanford, FL	29,900	83,100	38,100	45,000
Charlotte-Concord-Gastonia, NC-SC	25,400	89,700	36,500	53,200
Cincinnati, OH-KY-IN	28,700	93,600	37,600	56,000
Kansas City, MO-KS	23,200	83,800	34,400	49,400
Denver-Aurora-Lakewood, CO	21,200	73,800	32,800	41,000
Portland-Vancouver-Hillsboro, OR-WA	22,400	80,600	32,700	47,900
Sacramento-Roseville-Arden-Arcade, CA	21,400	75,800	32,900	42,900
Columbus, OH	22,100	83,100	39,300	43,800
Indianapolis-Carmel-Anderson, IN	19,300	77,200	34,000	43,200
Nashville-Davidson-Murfreesboro-Franklin, TN	18,900	76,000	35,700	40,300
Virginia Beach-Norfolk-Newport News, VA-NC	20,600	74,000	33,700	40,300
Las Vegas-Henderson-Paradise, NV	21,300	66,100	31,400	34,700
West Palm Beach, FL	31,000	57,500	26,500	31,000
Austin-Round Rock, TX	14,800	60,600	31,100	29,500
Memphis, TN-MS-AR	15,900	63,000	31,400	31,600
Providence-Warwick, RI-MA	18,100	62,000	26,000	36,000
Milwaukee-Waukesha-West Allis, WI	19,000	59,900	24,300	35,600
Jacksonville, FL	17,900	54,500	24,000	30,500
Oklahoma City, OK	12,200	58,400	23,700	34,700
Richmond, VA	16,600	54,100	23,900	30,200
Hartford-West Hartford-East Hartford, CT	14,000	45,000	19,800	25,200
Raleigh, NC	11,100	42,900	19,400	23,500
New Haven-Milford, CT	11,000	30,600	12,900	17,700
Salt Lake City, UT	8000	30,600	11,600	19,000
Southern NJ	**8300**	**20,400**	**9100**	**11,300**
Total (50 metro areas)	**2,041,000**	**6,327,300**	**2,810,200**	**3,517,100**
Relative to national totals	**52%**	**49%**	**51%**	**47%**

Note: highest and lowest MSA estimates are bolded.

**Table 3:** Diabetes population percentage metrics (ranked by diabetes cases), 2012.

Metropolitan area	Diabetes cases undiagnosed	Diagnosed diabetes			
		Uninsured	Insured		
			Type 2 diabetes	Type 2 diabetes	
				Treated with Rx	Treated
					Uncontrolled
New York-Newark-Jersey City, NY-NJ-PA (excluding Northern NJ)	29	**10**	93	70	44
Los Angeles-Long Beach-Anaheim, CA (excluding Orange County)	**37**	21	94	76	43
Chicago-Naperville-Elgin, IL-IN-WI	29	19	93	78	44
Northern NJ	29	13	91	71	45
Dallas-Fort Worth-Arlington, TX	28	23	93	80	47
Houston-The Woodlands-Sugar Land, TX	28	**25**	93	78	50
Philadelphia-Camden-Wilmington, PA-NJ-DE-MD	28	12	91	74	41
Miami-Fort Lauderdale, FL (excluding West Palm Beach, FL)	33	23	91	73	49
Washington-Arlington-Alexandria, DC-VA-MD-WV	29	16	91	77	47
Atlanta-Sandy Springs-Roswell, GA	27	21	93	78	46
Detroit-Warren-Dearborn, MI	26	14	94	70	46
San Francisco-Oakland-Hayward, CA	32	15	92	77	43
Boston-Cambridge-Newton, MA-NH	29	**9**	92	77	41
Phoenix-Mesa-Scottsdale, AZ	29	15	93	67	46
Riverside-San Bernardino-Ontario, CA	31	20	92	79	46
Tampa-St. Petersburg-Clearwater, FL	27	18	94	74	47
Seattle-Tacoma-Bellevue, WA	30	15	92	79	41
St. Louis, MO-IL	26	17	93	78	46
Orange County, CA	32	14	92	76	44
San Diego-Carlsbad, CA	32	18	92	78	39
Baltimore-Columbia-Towson, MD	28	11	93	75	48
Minneapolis-St. Paul-Bloomington, MN-WI	32	11	90	78	**36**
Pittsburgh, PA	28	11	93	76	41
Cleveland-Elyria, OH	**24**	14	92	77	43
San Antonio-New Braunfels, TX	27	**25**	**96**	78	**51**
Orlando-Kissimmee-Sanford, FL	28	21	93	74	46
Charlotte-Concord-Gastonia, NC-SC	26	21	94	78	41
Cincinnati, OH-KY-IN	**24**	16	92	77	40
Kansas City, MO-KS	26	18	93	78	41
Denver-Aurora-Lakewood, CO	32	16	**88**	78	44
Portland-Vancouver-Hillsboro, OR-WA	30	15	92	78	41
Sacramento-Roseville-Arden-Arcade, CA	32	15	93	78	43
Columbus, OH	25	16	92	79	47
Indianapolis-Carmel-Anderson, IN	26	19	91	80	44
Nashville-Davidson-Murfreesboro-Franklin, TN	**24**	20	92	80	47
Virginia Beach-Norfolk-Newport News, VA-NC	26	18	93	78	46
Las Vegas-Henderson-Paradise, NV	33	17	94	76	48
West Palm Beach, FL	29	13	90	**65**	46
Austin-Round Rock, TX	29	**24**	93	80	**51**
Memphis, TN-MS-AR	25	22	94	80	50
Providence-Warwick, RI-MA	29	12	92	77	42
Milwaukee-Waukesha-West Allis, WI	29	13	93	76	41
Jacksonville, FL	27	20	93	75	44
Oklahoma City, OK	25	22	94	**83**	41
Richmond, VA	25	18	92	77	44
Hartford-West Hartford-East Hartford, CT	31	11	93	76	44
Raleigh, NC	27	22	92	79	45
New Haven-Milford, CT	31	11	92	74	42
Salt Lake City, UT	31	14	89	79	38
Southern NJ	29	12	92	71	45
Total (50 metro areas)	**29**	**17**	**92**	**76**	**44**

Note: highest and lowest MSA estimates are bolded.

Among diabetes patients analyzed, 29% were undiagnosed; among diagnosed patients 17% were uninsured; among insured patients 92% had type 2 diabetes (Table 3). Los Angeles had the largest share of adults with undiagnosed diabetes (37%) as opposed to the lowest share in Cleveland Cincinnati, and Nashville (24%). A significant share of diagnosed diabetes patients lacked medical insurance, being the highest in three Texas MSAs (San Antonio [25%], Houston [25%], and Austin [24%]) and lowest in Boston (9%) and New York (10%). San Antonio had the highest share of insured, diagnosed patients that had type 2 diabetes (96%) in contrast to 88% in the Denver MSA.

### 3.2 Medication use and diabetes control

Among the insured population with type 2 diabetes, 76% had medication claims for antidiabetic agents—ranging from 83% in Oklahoma City to 65% in West Palm Beach (Table 3). Among those patients receiving medication, nationally 44% had medical claims indicating poorly controlled diabetes with local estimates lowest in Minneapolis (36%). MSAs in Texas ranked among the highest for patients with medical claims indicating uncontrolled diabetes—including Austin (51%), San Antonio (51%), Houston (50%), and Dallas-Fort Worth (47%).

## 4 Discussion

This study illustrates geographic variation in measures of diabetes prevalence, detection and management in 2012 and serves the following purposes: (1) providing a pre-ACA baseline to track outcomes over time, (2) providing communities with a comparison to state and national benchmarks, and (3) identifying communities performing well that could be used to identify best practices.

There is little published information at the metropolitan level for comparison to our findings. At the national level our results are similar to other estimates for the percentage of diagnosed diabetes that are type 2 and prevalence of undiagnosed diabetes. Our estimates of medication use based on claims data are lower than estimates based on self-reported data (CDC, 2013), while our estimates of patients receiving annual A1c testing and having their diabetes under control are similar to estimates based on electronic medical records (Courtemanche et al., 2013).

Geographic variation in diabetes care has been previously noted, but even with controlling for demographics, the source of the variation remains unclear (Egede et al., 2011a, Egede et al., 2011b; Ford et al., 2005; Lynch et al., 2015). Areas for future research include: (1) Understanding how expanded medical insurance coverage under the Affordable Care Act has helped close one gap to receiving treatment (i.e., high rates of uninsured people with diabetes); and (2) How new and expanded guidelines for screening asymptomatic adults for prediabetes and diabetes might help close another gap (i.e., high prevalence of undiagnosed diabetes).

### 4.1 Study strengths and limitations

The strengths of this study are use of large medical claims files covering the commercially insured, Medicare, and Medicaid populations to provide insight on treatment patterns, prevalence of diabetes-related complications, and health care expenditures. This analysis of medical claims provides a nice comparison to diabetes treatment and management statistics based on self-reported survey data collected through telephone survey (BRFSS) and suggests that self-reported data might overstate the proportion of patients receiving treatment for their diabetes.

Data limitations include the following:

• Claims data based case identification algorithms for type 2 diabetes may not be completely accurate. Algorithms that use a combination of both physician claims data and hospital discharge data have a sensitivity ranging from 57% to 95.6%, specificity ranging from 88% to 98.5%, positive predictive values ranging from 54% to 80%, and negative predictive values ranging from 98% to 99.6% (Khokhar et al., 2016). However, it has also been shown that more involved algorithms similar to ours that use a combination of physician claims, facility claims, and prescription drug claims and have multiple rules to differentiate type 1 from type 2 diabetes do have higher sensitivities (97% [95% CI 87–100]) for identifying type 1 diabetes and 93% [85–98] for type 2 diabetes (Klompas et al., 2013).
• For some MSAs, the medical claims sample was small for some demographic groups (in particular the age 20–34 population where diabetes prevalence is low). When the sample size fell below 30 adults for a particular demographic group, we then used information for that same demographic group at the next higher level of aggregation. For example, if the sample size for percent of diagnosed patients who were type 2 (versus type 1) was below 30 for a particular MSA, then we used the corresponding percentage at the state level for that demographic group. If the state sample size was below 30, then we used the corresponding percentage at the national level for that demographic group. Metropolitan-level estimates, therefore, were a combination of metropolitan, state and national metrics. This approach provides stability to estimates, but also biases the estimates towards the state and national averages.
• The medical claims analysis only includes insured patients in fee-for-service plans—as medical claims are often incomplete for patients in capitated managed care plans. It is unclear how this might bias study results. Medicare patients with poorer health might choose a fee-for-service plan because it gives them greater access to physicians not in a managed care network, but this would unlikely affect diabetes detection, treatment and control.
• Uncontrolled status was based on ICD-9 diagnosis codes and not by independent lab results. For validation, we analyzed the subset of commercially insured patients with type 2 diabetes for which we had both ICD-9 and A1c information. We found a significant and positive correlation between ICD-9 and A1c-based case identification measures (Spearman rank correlation coefficients of 0.22 [P < 0.001] for A1c > 9%). Patients who are sicker will tend to have more touch points with the health care system and thus generate more medical claims, so there is a higher likelihood that they might be categorized as uncontrolled diabetes. However, even with this limitation, ICD-9 diagnosis code-based definition for uncontrolled diabetes is still a valid and unbiased measure to be compared across geographic locations, especially if we have no reason to believe that physicians in different MSAs will have different tendency to use these diagnosis codes. Prior to the implementation of the ICD-10 system, Agency for Healthcare Research and Quality (AHRQ) diabetes quality measures included a measure on “uncontrolled diabetes admission rate” that also used ICD-9 codes to compare the quality of diabetes care across managed health plans (AHRQ, 2013).
• Medical claims data were unavailable for care veterans receive through the Veterans Health Administration and for patients in programs such as the Indian Health Service. For these government-sponsored programs adults age 65 or older are counted under Medicare, and adults under age 65 are counted under Medicaid.

## 5 Conclusion

This study used published information on the population and health characteristics of the population in the 50 largest metropolitan areas in the U.S. combined with analysis of medical claims files to estimate key metrics tracking access to care and treatment outcomes for people with diabetes (with the focus on type 2 diabetes). The study highlights that key diabetes prevalence and management metrics vary by MSA and can differ substantially from the national averages. Such information helps increase awareness of the areas where improvements can be made to inform strategies to improve diabetes screening and treatment, and tracked over time can help inform population health management strategies.

## Funding

Funding for this study was provided by Novo Nordisk Inc. Study co-author Erin Byrne (EB) is employed by Novo Nordisk Inc. and provided critical review and revision of the manuscript.

## Conflict of interest

The authors report the following competing interests: WY, TMD, ET, WI, RC, and FEL provide paid consulting services to pharmaceutical companies and other Life Sciences organizations. EB is employed by Novo Nordisk Inc.

## Appendix A Supplementary data

Supplementary tables Image 1
