# Supplementary material for: Diabetes diagnosis and management among insured adults across metropolitan areas in the U.S
Source: Prev Med Rep. 2018 Mar 28;10:227–33. doi: 10.1016/j.pmedr.2018.03.014 (PMC5984223; doi:10.1016/j.pmedr.2018.03.014)
Supplement: Supplementary file 1 — Supplementary tables [file mmc1.docx]

**Appendix**

Table A-1. 95% Confidence Intervals for Diabetes Population, Diagnosis and Insurance Status: Adults Age 20+, 2012

| **Metropolitan Area** | **Total Diabetes** | **# Undiagnosed Diabetes** | **Diagnosed** | |
| --- | --- | --- | --- | --- |
|  |  |  | **# Uninsured** | **# Insured** |
| New York-Newark-Jersey City, NY-NJ-PA (excluding Northern NJ) | ± 28,200 | ± 3,600 | ± 6,900 | ± 18,700 |
| Los Angeles-Long Beach-Anaheim, CA (excluding Orange County) | 16,300 | 2,200 | 5,300 | 8,900 |
| Chicago-Naperville-Elgin, IL-IN-WI | 22,800 | 2,800 | 7,900 | 14,100 |
| Northern NJ | 19,300 | 2,400 | 5,500 | 12,500 |
| Dallas-Fort Worth-Arlington, TX | 11,500 | 1,100 | 4,300 | 7,100 |
| Houston-The Woodlands-Sugar Land, TX | 11,300 | 1,200 | 4,500 | 6,900 |
| Philadelphia-Camden-Wilmington, PA-NJ-DE-MD | 14,000 | 1,600 | 3,700 | 9,400 |
| Miami-Fort Lauderdale, FL (excluding West Palm Beach, FL) | 12,800 | 1,900 | 4,700 | 7,200 |
| Washington-Arlington-Alexandria, DC-VA-MD-WV | 14,200 | 1,700 | 4,600 | 9,000 |
| Atlanta-Sandy Springs-Roswell, GA | 15,200 | 1,600 | 5,800 | 9,600 |
| Detroit-Warren-Dearborn, MI | 13,300 | 1,400 | 4,000 | 9,000 |
| San Francisco-Oakland-Hayward, CA | 7,000 | 900 | 1,900 | 4,400 |
| Boston-Cambridge-Newton, MA-NH | 13,300 | 1,300 | 3,300 | 8,900 |
| Phoenix-Mesa-Scottsdale, AZ | 15,100 | 1,400 | 4,400 | 9,900 |
| Riverside-San Bernardino-Ontario, CA | 5,900 | 700 | 2,100 | 3,600 |
| Tampa-St. Petersburg-Clearwater, FL | 5,900 | 600 | 2,000 | 3,800 |
| Seattle-Tacoma-Bellevue, WA | 11,700 | 1,200 | 3,400 | 7,400 |
| St. Louis, MO-IL | 8,400 | 900 | 2,900 | 5,500 |
| Orange County, CA | 3,900 | 300 | 1,000 | 2,400 |
| San Diego-Carlsbad, CA | 4,300 | 400 | 1,300 | 2,600 |
| Baltimore-Columbia-Towson, MD | 9,600 | 1,000 | 2,500 | 6,400 |
| Minneapolis-St. Paul-Bloomington, MN-WI | 12,900 | 1,400 | 3,300 | 8,100 |
| Pittsburgh, PA | 5,900 | 600 | 1,500 | 4,000 |
| Cleveland-Elyria, OH | 5,900 | 600 | 1,800 | 4,200 |
| San Antonio-New Braunfels, TX | 4,800 | 600 | 1,900 | 2,900 |
| Orlando-Kissimmee-Sanford, FL | 4,300 | 500 | 1,600 | 2,600 |
| Charlotte-Concord-Gastonia, NC-SC | 5,600 | 500 | 2,100 | 3,500 |
| Cincinnati, OH-KY-IN | 4,500 | 500 | 1,400 | 3,100 |
| Kansas City, MO-KS | 6,200 | 600 | 2,100 | 4,100 |
| Denver-Aurora-Lakewood, CO | 9,300 | 900 | 2,900 | 5,600 |
| Portland-Vancouver-Hillsboro, OR-WA | 7,800 | 800 | 2,400 | 5,000 |
| Sacramento--Roseville--Arden-Arcade, CA | 3,100 | 300 | 800 | 1,900 |
| Columbus, OH | 4,700 | 400 | 1,400 | 3,200 |
| Indianapolis-Carmel-Anderson, IN | 6,500 | 600 | 2,200 | 4,200 |
| Nashville-Davidson--Murfreesboro--Franklin, TN | 5,700 | 500 | 2,100 | 3,700 |
| Virginia Beach-Norfolk-Newport News, VA-NC | 5,400 | 700 | 1,900 | 3,500 |
| Las Vegas-Henderson-Paradise, NV | 11,000 | 1,300 | 3,400 | 6,600 |
| West Palm Beach, FL | 3,000 | 300 | 800 | 2,000 |
| Austin-Round Rock, TX | 3,000 | 300 | 1,100 | 1,800 |
| Memphis, TN-MS-AR | 5,300 | 600 | 2,100 | 3,400 |
| Providence-Warwick, RI-MA | 8,500 | 900 | 2,600 | 5,500 |
| Milwaukee-Waukesha-West Allis, WI | 8,300 | 800 | 2,300 | 5,400 |
| Jacksonville, FL | 2,700 | 300 | 1,000 | 1,700 |
| Oklahoma City, OK | 6,000 | 500 | 2,500 | 3,800 |
| Richmond, VA | 4,000 | 500 | 1,400 | 2,600 |
| Hartford-West Hartford-East Hartford, CT | 5,000 | 500 | 1,200 | 3,300 |
| Raleigh, NC | 3,100 | 300 | 1,200 | 2,000 |
| New Haven-Milford, CT | 3,600 | 400 | 900 | 2,400 |
| Salt Lake City, UT | 5,300 | 600 | 1,700 | 3,300 |
| Southern NJ | 1,000 | 100 | 300 | 700 |

**Table A-2. 95% Confidence Intervals for Diabetes T2 DM Population, Treatment Status, and Diabetes Control: Adults Age 20+, 2012**

| **Metropolitan Area** | **Type 2** | | | |
| --- | --- | --- | --- | --- |
|  | **# Untreated with Rx** | **Treated with Rx** | | |
|  |  | **# Treated** | **# Poorly controlled** | **# Controlled** |
| New York-Newark-Jersey City, NY-NJ-PA (excluding Northern NJ) | ±2,600 | ±2,600 | ±1,400 | ±1,400 |
| Los Angeles-Long Beach-Anaheim, CA (excluding Orange County) | 3,000 | 3,000 | 1,800 | 1,800 |
| Chicago-Naperville-Elgin, IL-IN-WI | 3,000 | 3,000 | 1,800 | 1,800 |
| Northern NJ | 2,000 | 2,000 | 1,100 | 1,100 |
| Dallas-Fort Worth-Arlington, TX | 2,000 | 2,000 | 1,300 | 1,300 |
| Houston-The Woodlands-Sugar Land, TX | 2,300 | 2,300 | 1,600 | 1,600 |
| Philadelphia-Camden-Wilmington, PA-NJ-DE-MD | 3,400 | 3,400 | 1,900 | 1,900 |
| Miami-Fort Lauderdale, FL (excluding West Palm Beach, FL) | 2,300 | 2,300 | 1,500 | 1,500 |
| Washington-Arlington-Alexandria, DC-VA-MD-WV | 2,300 | 2,300 | 1,400 | 1,400 |
| Atlanta-Sandy Springs-Roswell, GA | 2,200 | 2,200 | 1,300 | 1,300 |
| Detroit-Warren-Dearborn, MI | 3,400 | 3,400 | 2,200 | 2,200 |
| San Francisco-Oakland-Hayward, CA | 2,300 | 2,300 | 1,400 | 1,400 |
| Boston-Cambridge-Newton, MA-NH | 2,100 | 2,100 | 1,100 | 1,100 |
| Phoenix-Mesa-Scottsdale, AZ | 2,500 | 2,500 | 1,300 | 1,300 |
| Riverside-San Bernardino-Ontario, CA | 2,100 | 2,100 | 1,300 | 1,300 |
| Tampa-St. Petersburg-Clearwater, FL | 2,000 | 2,000 | 1,200 | 1,200 |
| Seattle-Tacoma-Bellevue, WA | 1,800 | 1,800 | 1,000 | 1,000 |
| St. Louis, MO-IL | 1,400 | 1,400 | 900 | 900 |
| Orange County, CA | 1,800 | 1,800 | 1,100 | 1,100 |
| San Diego-Carlsbad, CA | 1,800 | 1,800 | 900 | 900 |
| Baltimore-Columbia-Towson, MD | 1,500 | 1,500 | 1,000 | 1,000 |
| Minneapolis-St. Paul-Bloomington, MN-WI | 1,700 | 1,700 | 800 | 800 |
| Pittsburgh, PA | 3,400 | 3,400 | 1,900 | 1,900 |
| Cleveland-Elyria, OH | 1,800 | 1,800 | 1,000 | 1,000 |
| San Antonio-New Braunfels, TX | 1,700 | 1,700 | 1,100 | 1,100 |
| Orlando-Kissimmee-Sanford, FL | 1,400 | 1,400 | 800 | 800 |
| Charlotte-Concord-Gastonia, NC-SC | 1,300 | 1,300 | 700 | 700 |
| Cincinnati, OH-KY-IN | 1,500 | 1,500 | 800 | 800 |
| Kansas City, MO-KS | 1,400 | 1,400 | 800 | 800 |
| Denver-Aurora-Lakewood, CO | 1,700 | 1,700 | 900 | 900 |
| Portland-Vancouver-Hillsboro, OR-WA | 1,600 | 1,600 | 900 | 900 |
| Sacramento--Roseville--Arden-Arcade, CA | 1,700 | 1,700 | 1,000 | 1,000 |
| Columbus, OH | 1,400 | 1,400 | 900 | 900 |
| Indianapolis-Carmel-Anderson, IN | 1,300 | 1,300 | 800 | 800 |
| Nashville-Davidson--Murfreesboro--Franklin, TN | 1,600 | 1,600 | 1,000 | 1,000 |
| Virginia Beach-Norfolk-Newport News, VA-NC | 1,800 | 1,800 | 1,200 | 1,200 |
| Las Vegas-Henderson-Paradise, NV | 1,700 | 1,700 | 1,100 | 1,100 |
| West Palm Beach, FL | 1,200 | 1,200 | 700 | 700 |
| Austin-Round Rock, TX | 1,200 | 1,200 | 800 | 800 |
| Memphis, TN-MS-AR | 1,200 | 1,200 | 800 | 800 |
| Providence-Warwick, RI-MA | 1,000 | 1,000 | 500 | 500 |
| Milwaukee-Waukesha-West Allis, WI | 1,000 | 1,000 | 500 | 500 |
| Jacksonville, FL | 1,100 | 1,100 | 600 | 600 |
| Oklahoma City, OK | 1,000 | 1,000 | 600 | 600 |
| Richmond, VA | 1,100 | 1,100 | 600 | 600 |
| Hartford-West Hartford-East Hartford, CT | 1,000 | 1,000 | 500 | 500 |
| Raleigh, NC | 900 | 900 | 600 | 600 |
| New Haven-Milford, CT | 500 | 500 | 300 | 300 |
| Salt Lake City, UT | 800 | 800 | 400 | 400 |
| Southern NJ | 500 | 500 | 300 | 300 |

Table A-3. 95% Confidence Intervals (± Percentage Point) for Diabetes Population Percentage Metrics, 2012

| **Metropolitan Area** | **% Diabetes Cases Undiagnosed** | **Diagnosed Diabetes** | | | |
| --- | --- | --- | --- | --- | --- |
|  |  | **% Uninsured** | **Insured** | | |
|  |  |  | **%**  **Type 2 Diabetes** | **Type 2 Diabetes** | |
|  |  |  |  | **% Treated with Rx** | **Treated** |
|  |  |  |  |  | **% Uncontrolled** |
| New York-Newark-Jersey City, NY-NJ-PA (excluding Northern NJ) | ± 0.3% | ± 0.6% | ± 0.2% | ± 0.5% | ± 0.6% |
| Los Angeles-Long Beach-Anaheim, CA (excluding Orange County) | 0.2% | 0.5% | 0.4% | 0.8% | 1.1% |
| Chicago-Naperville-Elgin, IL-IN-WI | 0.3% | 0.9% | 0.4% | 0.8% | 1.2% |
| Northern NJ | 0.4% | 0.9% | 0.4% | 0.8% | 1.0% |
| Dallas-Fort Worth-Arlington, TX | 0.2% | 0.7% | 0.4% | 0.9% | 1.2% |
| Houston-The Woodlands-Sugar Land, TX | 0.2% | 0.8% | 0.5% | 1.0% | 1.4% |
| Philadelphia-Camden-Wilmington, PA-NJ-DE-MD | 0.3% | 0.7% | 0.8% | 1.4% | 2.0% |
| Miami-Fort Lauderdale, FL (excluding West Palm Beach, FL) | 0.3% | 0.9% | 0.7% | 1.3% | 1.7% |
| Washington-Arlington-Alexandria, DC-VA-MD-WV | 0.3% | 0.9% | 0.5% | 1.0% | 1.4% |
| Atlanta-Sandy Springs-Roswell, GA | 0.3% | 1.2% | 0.4% | 1.0% | 1.4% |
| Detroit-Warren-Dearborn, MI | 0.3% | 0.9% | 0.9% | 1.9% | 2.7% |
| San Francisco-Oakland-Hayward, CA | 0.2% | 0.5% | 0.8% | 1.4% | 1.9% |
| Boston-Cambridge-Newton, MA-NH | 0.3% | 0.8% | 0.9% | 1.1% | 1.5% |
| Phoenix-Mesa-Scottsdale, AZ | 0.4% | 1.2% | 0.6% | 1.7% | 1.9% |
| Riverside-San Bernardino-Ontario, CA | 0.2% | 0.6% | 0.8% | 1.4% | 2.0% |
| Tampa-St. Petersburg-Clearwater, FL | 0.2% | 0.7% | 0.7% | 1.6% | 2.0% |
| Seattle-Tacoma-Bellevue, WA | 0.4% | 1.1% | 0.9% | 1.4% | 1.9% |
| St. Louis, MO-IL | 0.3% | 1.1% | 0.6% | 1.2% | 1.6% |
| Orange County, CA | 0.1% | 0.4% | 0.9% | 1.7% | 2.2% |
| San Diego-Carlsbad, CA | 0.2% | 0.5% | 0.9% | 1.7% | 2.2% |
| Baltimore-Columbia-Towson, MD | 0.4% | 1.0% | 0.7% | 1.3% | 1.8% |
| Minneapolis-St. Paul-Bloomington, MN-WI | 0.6% | 1.4% | 0.7% | 1.6% | 2.1% |
| Pittsburgh, PA | 0.3% | 0.7% | 1.4% | 3.2% | 4.3% |
| Cleveland-Elyria, OH | 0.3% | 0.8% | 1.0% | 1.7% | 2.3% |
| San Antonio-New Braunfels, TX | 0.3% | 0.8% | 0.8% | 1.8% | 2.4% |
| Orlando-Kissimmee-Sanford, FL | 0.2% | 0.8% | 0.8% | 1.7% | 2.2% |
| Charlotte-Concord-Gastonia, NC-SC | 0.2% | 1.0% | 0.8% | 1.4% | 1.9% |
| Cincinnati, OH-KY-IN | 0.2% | 0.7% | 0.7% | 1.6% | 2.0% |
| Kansas City, MO-KS | 0.3% | 1.1% | 0.7% | 1.7% | 2.3% |
| Denver-Aurora-Lakewood, CO | 0.5% | 1.5% | 1.0% | 2.2% | 2.9% |
| Portland-Vancouver-Hillsboro, OR-WA | 0.4% | 1.3% | 1.2% | 2.0% | 2.7% |
| Sacramento--Roseville--Arden-Arcade, CA | 0.2% | 0.4% | 1.2% | 2.2% | 2.9% |
| Columbus, OH | 0.2% | 0.8% | 0.8% | 1.7% | 2.4% |
| Indianapolis-Carmel-Anderson, IN | 0.3% | 1.3% | 1.1% | 1.6% | 2.3% |
| Nashville-Davidson--Murfreesboro--Franklin, TN | 0.3% | 1.2% | 1.2% | 2.1% | 2.9% |
| Virginia Beach-Norfolk-Newport News, VA-NC | 0.4% | 1.1% | 1.4% | 2.4% | 3.5% |
| Las Vegas-Henderson-Paradise, NV | 0.8% | 2.0% | 1.1% | 2.6% | 3.4% |
| West Palm Beach, FL | 0.2% | 0.5% | 1.1% | 2.0% | 2.5% |
| Austin-Round Rock, TX | 0.2% | 0.7% | 0.9% | 2.0% | 2.7% |
| Memphis, TN-MS-AR | 0.4% | 1.5% | 1.0% | 1.9% | 2.7% |
| Providence-Warwick, RI-MA | 0.6% | 1.9% | 0.9% | 1.5% | 2.0% |
| Milwaukee-Waukesha-West Allis, WI | 0.6% | 1.7% | 0.7% | 1.6% | 2.1% |
| Jacksonville, FL | 0.2% | 0.8% | 1.1% | 2.1% | 2.7% |
| Oklahoma City, OK | 0.4% | 1.9% | 0.8% | 1.7% | 2.4% |
| Richmond, VA | 0.4% | 1.1% | 1.1% | 2.0% | 2.6% |
| Hartford-West Hartford-East Hartford, CT | 0.5% | 1.2% | 1.2% | 2.1% | 2.7% |
| Raleigh, NC | 0.3% | 1.2% | 1.3% | 2.2% | 3.1% |
| New Haven-Milford, CT | 0.5% | 1.2% | 0.8% | 1.7% | 2.1% |
| Salt Lake City, UT | 0.8% | 2.3% | 1.7% | 2.5% | 3.4% |
| Southern NJ | 0.2% | 0.6% | 1.2% | 2.2% | 3.0% |
